# *Rhodobacter capsulatus* AnfA is essential for production of Fe‐nitrogenase proteins but dispensable for cofactor biosynthesis and electron supply

**DOI:** 10.1002/mbo3.1033

**Published:** 2020-03-23

**Authors:** Lisa Demtröder, Yvonne Pfänder, Bernd Masepohl

**Affiliations:** ^1^ Microbial Biology Faculty of Biology and Biotechnology Ruhr University Bochum Bochum Germany

**Keywords:** AnfA, Fe‐nitrogenase, Mo‐nitrogenase, NifA, *Rhodobacter*

## Abstract

The photosynthetic α‐proteobacterium *Rhodobacter capsulatus* reduces and thereby fixes atmospheric dinitrogen (N_2_) by a molybdenum (Mo)‐nitrogenase and an iron‐only (Fe)‐nitrogenase. Differential expression of the structural genes of Mo‐nitrogenase (*nifHDK*) and Fe‐nitrogenase (*anfHDGK*) is strictly controlled and activated by NifA and AnfA, respectively. In contrast to NifA‐binding sites, AnfA‐binding sites are poorly defined. Here, we identified two highly similar AnfA‐binding sites in the *R. capsulatus anfH* promoter by studying the effects of promoter mutations on in vivo* anfH* expression and in vitro promoter binding by AnfA. Comparison of the experimentally determined *R. capsulatus* AnfA‐binding sites and presumed AnfA‐binding sites from other α‐proteobacteria revealed a consensus sequence of dyad symmetry, TAC–N_6_–GTA, suggesting that AnfA proteins bind their target promoters as dimers. Chromosomal replacement of the *anfH* promoter by the *nifH* promoter restored *anfHDGK* expression and Fe‐nitrogenase activity in an *R. capsulatus* strain lacking AnfA suggesting that AnfA is required for AnfHDGK production, but dispensable for biosynthesis of the iron‐only cofactor and electron delivery to Fe‐nitrogenase, pathways activated by NifA. These observations strengthen our model, in which the Fe‐nitrogenase system in *R. capsulatus* is largely integrated into the Mo‐nitrogenase system.

## Graphical abstract

*Rhodobacter capsulatus* fixes nitrogen from air by a molybdenum‐dependent and an iron‐only nitrogenase. Here, we present evidence that the transcriptional activator AnfA is required for AnfHDGK production, but dispensable for biosynthesis of the Fe‐only cofactor and electron delivery.

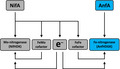

## 1 INTRODUCTION

Molecular nitrogen (N_2_) is highly abundant in air, but only diazotrophic bacteria and archaea are capable of utilizing this source of nitrogen, while non‐diazotrophic prokaryotes and all eukaryotes depend on more reduced nitrogen forms like ammonium, nitrate, or organic nitrogen compounds (Dos Santos, Fang, Mason, Setubal, & Dixon, 2012; Zhang & Gladyshev, 2008; Zhang, Rump, & Gladyshev, 2011). In a process called biological nitrogen fixation, diazotrophs reduce N_2_ to ammonia (NH_3_) by three structurally and functionally similar nitrogenase isoenzymes, each composed of two components, a catalytic dinitrogenase and a dinitrogenase reductase. All diazotrophs possess a molybdenum nitrogenase consisting of the NifDK and NifH components. Also, some diazotrophs encode either a vanadium nitrogenase (VnfDGK, VnfH) or an iron‐only nitrogenase (AnfDGK, AnfH), or both Mo‐free isoenzymes (McRose, Zhang, Kraepiel, & Morel, 2017). Since Mo‐nitrogenase is more efficient than the other nitrogenases in terms of ATP consumption per N_2_ reduced, diazotrophs preferentially synthesize Mo‐nitrogenase when ammonium is limiting (Eady, 2003; Mus, Alleman, Pence, Seefeldt, & Peters, 2018; Schneider, Gollan, Dröttboom, Selsemeier‐Voigt, & Müller, 1997; Sippel & Einsle, 2017; Thiel & Pratte, 2014). Many diazotrophs repress the production of Mo‐free nitrogenases by one‐component ModE regulators, which directly sense and respond to molybdate availability (reviewed by Demtröder, Narberhaus, & Masepohl, 2019). The Mo‐free nitrogenases take over under Mo‐limiting conditions.

Mo‐nitrogenase contains a complex iron‐molybdenum cofactor, FeMoco, whose synthesis requires the NifUS, NifH, NifB, NifEN, NifQ, and NifV proteins (reviewed by Burén, Jiménez‐Vicente, Echavarri‐Erasun, & Rubio, 2020; Curatti & Rubio, 2014; Hu & Ribbe, 2016). The NifUS, NifB, and NifV proteins are also involved in biosynthesis of the iron‐vanadium cofactor of V‐nitrogenase, FeVco, and the iron‐only cofactor of Fe‐nitrogenase, FeFeco (Drummond, Walmsley, & Kennedy, 1996; Hamilton et al., 2011; Hu & Ribbe, 2016; Kennedy & Dean, 1992; Sippel & Einsle, 2017; Yang, Xie, Wang, Dixon, & Wang, 2014). Electron transfer to the nitrogenases involves species‐specific proteins like NifF, FdxN, RnfABCDGEH, and FixABC (Boyd, Costas, Hamilton, Mus, & Peters, 2015; Dos Santos et al., 2012; Oldroyd, 2013; Poudel et al., 2018).

**FIGURE A1:**
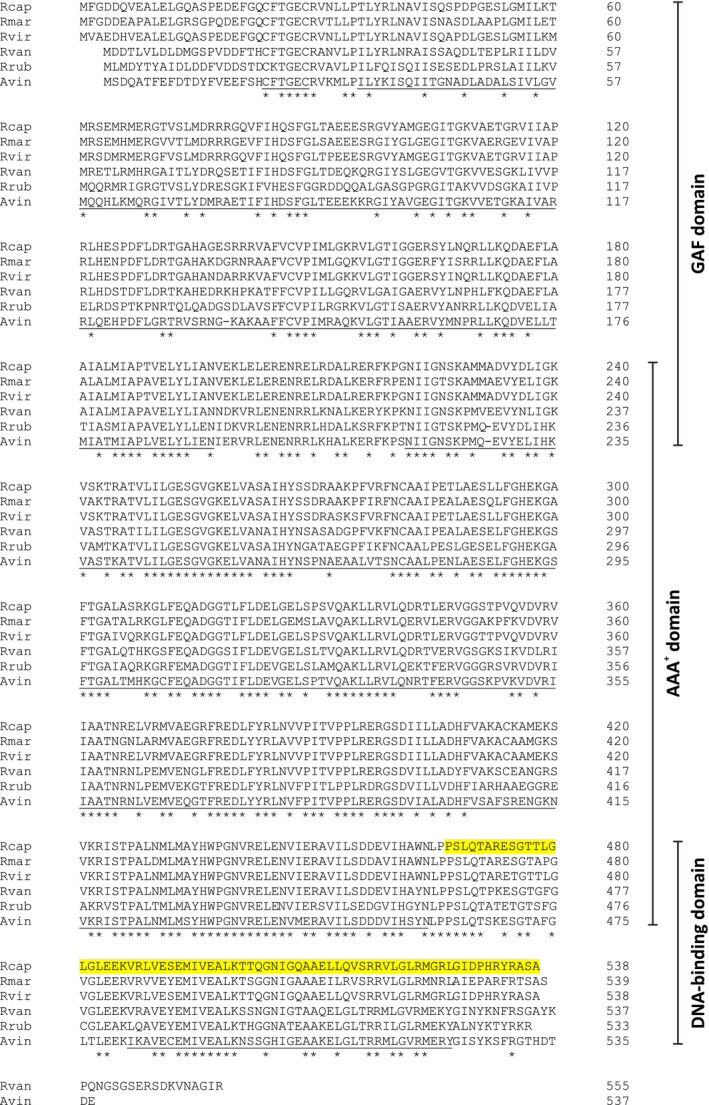
Comparison of AnfA proteins

In proteobacteria, expression of *nif*, *vnf*, and *anf* genes depends on the alternative sigma factor RpoN and the structurally and functionally related transcription activators NifA, VnfA, and AnfA, respectively (Bush & Dixon, 2012; Dixon & Kahn, 2004; Drummond et al., 1996; Fischer, 1994; Hamilton et al., 2011; Heiniger, Oda, Samanta, & Harwood, 2012; Hübner, Masepohl, Klipp, & Bickle, 1993; Joerger, Jacobson, & Bishop, 1989; Kutsche, Leimkühler, Angermüller, & Klipp, 1996; Merrick, 1993; Mus et al., 2018; Oda et al., 2005; Oliveira et al., 2012; Sarkar & Reinhold‐Hurek, 2014; Souza, Pedrosa, Rigo, Machado, & Yates, 2000; Walmsley, Toukdarian, & Kennedy, 1994; Zhang, Pohlmann, Ludden, & Roberts, 2000; Zou et al., 2008). These activators encompass three modules, namely an N‐terminal GAF, a central AAA+, and a C‐terminal HTH domain, involved in environmental sensing, ATP‐dependent activation of target gene transcription, and DNA binding, respectively. Many NifA proteins contain a conserved cysteine motif in an interdomain linker connecting the central and C‐terminal domains, while VnfA and AnfA proteins contain conserved cysteine motifs in their GAF domains (Figure A1 in Appendix 2). The cysteine residues are known or presumed to bind iron‐sulfur clusters making these proteins sensitive to oxygen (Austin & Lambert, 1994; Fischer, 1994; Nakajima et al., 2010; Yoshimitsu, Takatani, Miura, Watanabe, & Nakajima, 2011). This prevents inappropriate induction of nitrogen fixation, a process not compatible with oxygen.

RpoN proteins bind promoters with the consensus CTGG–N_8_–TTGC (N stands for any nucleotide) located at position –24/–12 upstream of the transcription start site (Buck, Miller, Drummond, & Dixon, 1986; Bush & Dixon, 2012; Fischer, 1994; Merrick, 1993; Zhang & Buck, 2015). NifA proteins bind cis‐regulatory elements with the consensus TGT–N_10_–ACA, which is typically found within a distance of 150 base‐pairs upstream of the transcription start site (Buck et al., 1986; Demtröder, Pfänder, Schäkermann, Bandow, & Masepohl, 2019; Fischer, 1994). NifA‐binding sites are well‐characterized in many α‐, β‐, and γ‐proteobacteria (Barrios, Grande, Olvera, & Morett, 1998; Buck et al., 1986; González, Olvera, Sobero, & Morett, 1998; Gubler, 1989; Lee, Berger, & Kustu, 1993; Monteiro et al., 1999; Wang, Kolb, Cannon, & Buck, 1997), whereas VnfA‐ and AnfA‐binding sites have been studied so far only in the γ‐proteobacterium *Azotobacter vinelandii*, which is one of few diazotrophs having both Mo‐free nitrogenases (Austin & Lambert, 1994; Drummond et al., 1996; Frise, Green, & Drummond, 1994; Woodley, Buck, & Kennedy, 1996). In this species, the sequences GTAC–N_6_–GTAC and C–N–GG–N_3_–GGTA have been suggested as binding sites for VnfA and AnfA, respectively (Austin & Lambert, 1994; Woodley et al., 1996).

**FIGURE 1:**
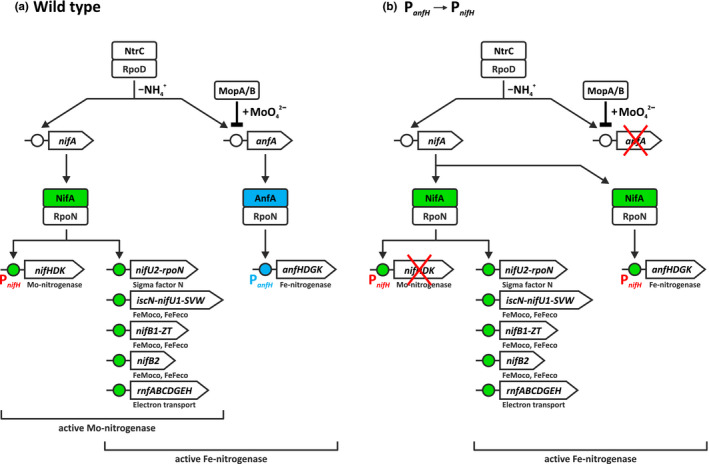
Model of the nitrogen fixation regulon in *Rhodobacter capsulatus*. (a) Production of Mo‐ and Fe‐nitrogenases in the wild type. In the absence of ammonium (–NH_4_^+^), the superior regulator NtrC activates transcription of *nifA* and *anfA* in concert with the housekeeping sigma factor RpoD (Foster‐Hartnett, Cullen, Monika, & Kranz, 1994; Kutsche et al., 1996). MopA and MopB independently repress *anfA* in the presence of molybdate (+MoO_4_^2–^; Wiethaus et al., 2006). NifA and AnfA activate their target genes by partnering with the alternative sigma factor RpoN. Noteworthy, NifA indirectly controls AnfA‐mediated *anfHDGK* expression by controlling RpoN production (Demtröder, Pfänder, et al., 2019). Involvement of NifA‐activated genes in biosynthesis of the iron‐molybdenum cofactor (FeMoco) of Mo‐nitrogenase and the iron‐only cofactor (FeFeco) of Fe‐nitrogenase and electron transfer to both nitrogenases is indicated. (b) Production of active Fe‐nitrogenase in a strain lacking AnfA. In this study, we constructed strain YP515‐BS85 containing mutations in the *anfA* and *nifD* genes (marked by red crosses) and a chromosomal substitution of the *anfH* promoter (P*_anfH_*) by the *nifH* promoter (P*_nifH_*) thereby putting *anfHDGK* expression under NifA control. This strain grew under N_2_‐fixing conditions (Figure 4b) suggesting that AnfA is dispensable for FeFeco biosynthesis and electron supply to Fe‐nitrogenase. For further details, see text

To determine AnfA‐binding site requirements in a diazotroph distantly related to *A. vinelandii*, we examined the regulation of Fe‐nitrogenase genes in the photosynthetic α‐proteobacterium *Rhodobacter capsulatus*, which fixes nitrogen by the Mo‐ and Fe‐nitrogenases (Schneider, Müller, Schramm, & Klipp, 1991; Schüddekopf, Hennecke, Liese, Kutsche, & Klipp, 1993; Strnad et al., 2010). Differential expression of the corresponding *nif* and *anf* genes is tightly regulated. While NifA activates the promoters upstream of multiple *nif* genes, AnfA activates the *anfH* promoter as indicated by reporter fusions and proteome profiling (Demtröder, Pfänder, et al., 2019; Figure 1a). Since the sigma factor RpoN is encoded by the *nifU2*‐*rpoN* operon, NifA indirectly controls AnfA‐mediated expression of the Fe‐nitrogenase genes (Demtröder, Pfänder, et al., 2019).

In this study, we show that *R. capsulatus* AnfA binds two highly similar palindromic sites in the *anfH* promoter. Based on conserved sequences in various α‐proteobacteria, we define a general AnfA‐binding site consensus, TAC–N_6_–GTA. Besides, we present evidence that the *anfH* promoter is the only Fe‐nitrogenase‐related promoter in *R. capsulatus* strictly depending on AnfA.

## 2 MATERIALS AND METHODS

### 2.1 Strains, plasmids, and growth conditions

Bacterial strains and plasmids used in this study are listed in Table A1 in Appendix 1. *Rhodobacter capsulatus* minimal medium V (RCV) was prepared as previously described (Demtröder, Pfänder, et al., 2019). In this medium, a fixed nitrogen source and molybdate (Mo) have been omitted. Traces of Mo arising from impurities of the chemicals used support residual Mo‐nitrogenase activity but are low enough to permit the production of Fe‐nitrogenase. To examine diazotrophic growth, cultures were inoculated in 3 ml RCV medium in screw‐capped 17‐ml Hungate tubes before the exchange of headspace air for pure N_2_ gas and incubation in the light. When required, 10 mM serine was added as a fixed nitrogen source, which (in contrast to ammonium) does not inhibit nitrogen fixation.

### 2.2 Construction of *Rhodobacter capsulatus anfH‐lacZ* reporter strains and β‐galactosidase assays

**FIGURE 2:**
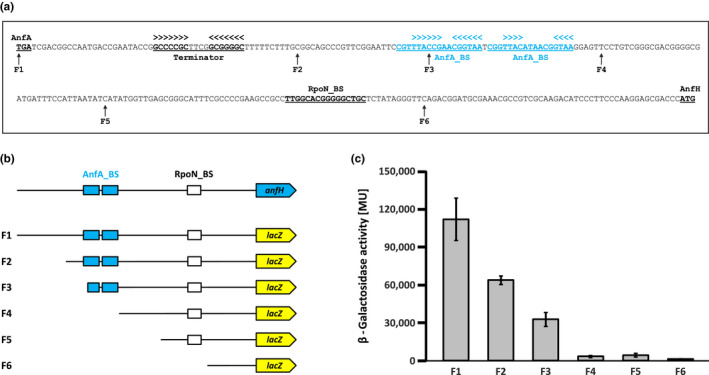
Effect of nested deletions in the *R. capsulatus anfH* promoter on *anfH‐lacZ* expression. (a) Cis‐regulatory elements in the *anfA*‐*anfH* intergenic region. The DNA sequence encompasses the AnfA translation stop codon (TGA), the Rho‐independent *anfA* transcription terminator, two AnfA‐binding sites (AnfA_BS), the RpoN‐binding site (RpoN_BS), and the AnfH translation start codon (ATG). Arrowheads mark inverted repeat sequences. The start sites of *anfH* promoter deletion variants F1 to F6 are indicated. (b) Reporter fusions between *anfH* promoter deletion variants and *lacZ*. Promoter variants F1 to F6 were cloned into a broad‐host‐range vector, before insertion of a *lacZ* cassette (designed for transcriptional fusions) immediately downstream of the *anfH* start codon resulting in reporter plasmids pBBR_F1‐*lacZ* to pBBR_F6‐*lacZ* (Materials and Methods). (c) Expression of *anfH*‐*lacZ* fusions. *R. capsulatus* reporter strains carrying pBBR_F1‐*lacZ* to pBBR_F6‐*lacZ* were phototrophically grown in RCV minimal medium with 10 mM serine but without Mo addition, conditions allowing *anfHDGKOR3* expression. LacZ (β‐galactosidase) activity is given in Miller units (Miller, 1972). The results represent the means and standard deviations of five independent experiments

The *anfH* promoter was narrowed down by nested deletions. For this, appropriate primer pairs were used to PCR‐amplify promoter variants F1 to F6 (Figure 2a,b), thereby adding BamHI and HindIII sites. Corresponding BamHI‐HindIII fragments were cloned into the broad‐host‐range vector pBBR1MCS (Kovach et al., 1995) before insertion of a *lac*TeT cassette (carrying a promoterless *lacZ* gene, a tetracycline resistance gene, and an oriT transfer origin) from plasmid pYP35 (Gisin et al., 2010) into the HindIII site. The resulting reporter plasmids carrying transcriptional *lacZ* fusions were designated pBBR_F1‐*lacZ* to pBBR_F6‐*lacZ*.

**FIGURE 3:**
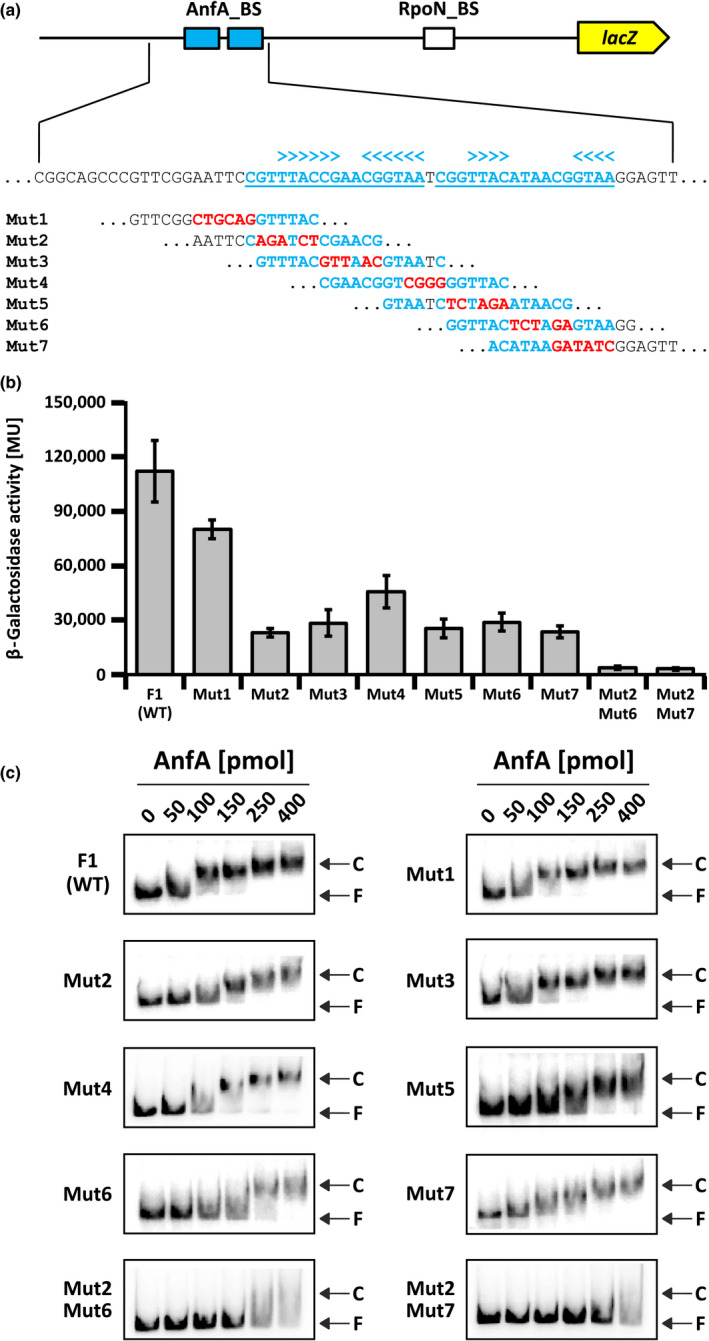
Effect of base substitutions in the *anfH* promoter on *anfH‐lacZ* expression and AnfA binding. (a) Base substitutions in the AnfA‐binding sites. Plasmid pBBR_F1‐*lacZ* (carrying the wild‐type *anfH* promoter fragment F1 fused to *lacZ* shown in Figure 2b) served as a template for base substitution mutations Mut1 to Mut7 (highlighted in red). (b) Expression of *anfH*‐*lacZ* fusions. *R. capsulatus* reporter strains carrying pBBR_F1‐*lacZ* (WT) and its variants (Mut1 to Mut7, Mut2/6, and Mut2/7) were phototrophically grown in RCV minimal medium (no Mo added) with 10 mM serine. LacZ (β‐galactosidase) activity is given in Miller units (Miller, 1972). Data for WT control are the same as in Figure 2c. The results represent the means and standard deviations of five independent experiments. (c) Binding of AnfA_DBD to the *anfH* promoter. In vitro binding of the separated DNA‐binding domain of AnfA (AnfA_DBD) to the *anfH* promoter was examined by EMSA. PCR fragments carrying the wild‐type (WT; F1 fragment) *anfH* promoter and its variants Mut1 to Mut7, Mut2/6, and Mut2/7 were ^32^P‐labeled before incubation with the indicated amounts of AnfA protein. AnfA‐promoter complexes and free promoter fragments (labeled C and F, respectively) were electrophoretically separated and detected by autoradiography. EMSA analyses were done in duplicate with one representative result shown in (c)

To generate site‐directed substitution mutations in the *anfH* promoter (Figure 3a), plasmid pBBR_F1‐*lacZ* served as a template. Base‐pair substitutions were introduced using the QuikChange protocol (Stratagene). The resulting pBBR_F1‐*lacZ* derivatives carrying transcriptional *lacZ* fusions to different promoter variants were designated pBBR_Mut1‐*lacZ* to pBBR_Mut7‐*lacZ*, pBBR_Mut2/6‐*lacZ*, and pBBR_Mut2/7‐*lacZ*.

The reporter plasmids were conjugationally transferred into the *R. capsulatus* wild‐type strain B10S. Following phototrophic growth of the *R. capsulatus* reporter strains in RCV medium with 10 mM serine (no Mo added) until the late logarithmic phase, LacZ (β‐galactosidase) activity was determined (Miller, 1972).

### 2.3 Examination of AnfA binding to the *anfH* promoter

In vitro binding of the DNA‐binding domain of AnfA (AnfA_DBD) to the *anfH* promoter was examined by electrophoretic mobility shift assays (EMSA) as previously described (Müller et al., 2010). To overexpress AnfA_DBD, appropriate primers were used to PCR‐amplify a DNA fragment coding for the C‐terminal 72 amino acid residues of AnfA (Figure A1 in Appendix 2) thereby adding SacII and NcoI sites. The corresponding SacII‐NcoI fragment was cloned into the expression vector pASK_IBA45+ (IBA GmbH Göttingen) resulting in hybrid plasmid pYP409. For purification of the Strep‐tagged AnfA_DBD, *Escherichia coli* BL21 (DE) carrying pYP409 was cultivated with AHT induction before cell disruption and Strep‐Tactin affinity chromatography as previously described (Hoffmann, Ali, et al., 2016).

The F1 fragment (carrying the wild‐type *anfH* promoter) and its variants (Figure 3a) were labeled with γ‐^32^P‐ATP, and free γ‐^32^P‐ATP was removed by gel filtration using Illustra ProbeQuant G‐50 Micro Columns (GE‐Healthcare). After 20 min incubation of labeled promoter variants with increasing amounts of the purified AnfA_DBD protein, bound and free DNAs were separated in 6% polyacrylamide gels. Radioactive bands were detected by phosphor screen exposure.

### 2.4 Substitution of the *Rhodobacter capsulatus anfH* promoter by the *nifH* promoter

To replace the *anfH* promoter (P*_anfH_*) by the *nifH* promoter (P*_nifH_*), we exchanged the 266 bp *anfA*‐*anfH* intergenic region by the 267 bp *fdxD*‐*nifH* intergenic region. For this purpose, we constructed mutagenesis plasmid pYP516 containing the 3′ end of *anfA* (including the translation stop codon, TGA), a gentamicin (Gm) resistance cassette, P*_nifH_*, and the 5′ end of *anfH* (starting with the translation start codon, ATG). To replace the *anfH* promoter and to delete the *anfA* gene in a single step, we constructed mutagenesis plasmid pYP515 containing an *anfA* upstream fragment (but lacking the *anfA* coding region), a Gm cassette, P*_nifH_*, and the 5′ end of *anfH*. Plasmids pYP516 and pYP515 were conjugationally introduced into the *R. capsulatus* strain BS85 (Δ*nifD*), in which the *nifD* gene is disrupted by a spectinomycin (Sp) cassette. BS85 does not exhibit Mo‐nitrogenase activity, and hence, any nitrogenase activity observed in this background can be assigned to Fe‐nitrogenase (Demtröder, Pfänder, et al., 2019). Promoter replacement mutants were identified by selection for Gm resistance and screening for loss of vector‐encoded tetracycline resistance indicating marker rescue by double cross‐over events. The resulting mutants were called YP516‐BS85 (P*_anfH_* → P*_nifH_*, *anfA* ^+^, Δ*nifD*) and YP515‐BS85 (P*_anfH_* → P*_nifH_*, Δ*anfA*, Δ*nifD*).

In contrast to strain YP515‐BS85 (P*_anfH_* → P*_nifH_*, Δ*anfA*, Δ*nifD*), strain KS94A‐YP415 (Δ*anfA*, Δ*nifD*) contains the wild‐type *anfH* promoter upstream of the *anfHDGKOR3* operon. In this strain, the *anfA* and *nifD* genes are disrupted by Sp and Gm cassettes, respectively.

## 3 RESULTS

### 3.1 Localization of the *Rhodobacter capsulatus anfH* promoter by nested deletions

*Rhodobacter capsulatus* AnfA is essential for the expression of the *anfHDGKOR3* operon (Demtröder, Pfänder, et al., 2019), but the *anfH* promoter has not been investigated. The coding regions of *anfH* and its upstream gene, *anfA*, are separated by 266 bp (Figure 2a). This intergenic region includes three conspicuous sequences, namely (a) a GC‐rich inverted repeat sequence followed by a T‐rich stretch likely acting as Rho‐independent terminator of *anfA* transcription, (b) two 17 bp direct repeats each encompassing inverted repeat sequences, which are promising candidates as AnfA‐binding sites, and (c) a highly conserved RpoN‐binding site. For clarity, the 17 bp sequences will from now on be called distal and proximal AnfA‐binding sites.

To localize the *anfH* promoter (P*_anfH_*), we analyzed the effects of nested promoter deletions on *anfH* expression. For this purpose, we generated transcriptional fusions between P*_anfH_* fragments, F1 to F6, and a promoterless *lacZ* gene (Figure 2b). *R. capsulatus* strains carrying the corresponding reporter plasmids were grown in RCV minimal medium with serine as a nitrogen source without molybdate addition, conditions compatible with the synthesis of Fe‐nitrogenase (Demtröder, Pfänder, et al., 2019; Hoffmann, Wagner, et al., 2016), before determination of LacZ (β‐galactosidase) activity.

Fragments F1 and F2 mediated considerable LacZ activity (Figure 2c) indicating that the *anfA*‐*anfH* intergenic region and in particular the region downstream of the putative *anfA* transcription terminator contain all cis‐regulatory elements required for P*_anfH_* activation. F3‐based LacZ activity was reduced to 29% of the F1 value probably due to the absence of one half‐site of the distal AnfA‐binding site (see below). As expected, fragments F4 to F6 resulted in only background LacZ activity consistent with the absence of both AnfA‐binding sites.

### 3.2 Effects of AnfA‐binding site mutations on *anfH* expression

To dissect the function of the distal and proximal AnfA‐binding sites in P*_anfH_*, we generated pBBR_F1‐*lacZ* variants carrying site‐directed mutations Mut1 to Mut7 (Figure 3a) and the combinations Mut2/6 and Mut2/7. Mut1 mediated clear LacZ activity (72% of the F1 value) suggesting that this sequence plays only a minor role in *anfH* expression consistent with our findings on nested promoter deletions (Figure 2). LacZ activity of Mut2 to Mut7 dropped to 20%–40% as compared to F1 (carrying the wild‐type *anfH* promoter), while combined mutations Mut2/6 and Mut2/7 abolished LacZ activity (Figure 3b). These observations suggest that either of the two AnfA‐binding sites mediates P*_anfH_* activation to some extent and that full activation requires both sites.

### 3.3 Effects of AnfA‐binding site mutations on promoter binding by AnfA

The AnfA protein encompasses three domains, namely a GAF, an AAA+, and a HTH domain, involved in environmental sensing, activation of RNA polymerase, and promoter binding, respectively. To test the direct binding of AnfA to P*_anfH_*, we performed electrophoretic mobility shift assays (EMSA). For this, the radiolabeled F1 fragment (carrying the wild‐type *anfH* promoter) and its variants (Mut1 to Mut7, Mut2/6, and Mut2/7) were incubated with increasing amounts of the Strep‐tagged DNA‐binding domain of AnfA (AnfA_DBD) encompassing the C‐terminal 72 amino acid residues of the full‐length regulator (Figure A1 in Appendix 2). AnfA_DBD was expected to bind the same target sequence as the full‐length regulator, but to be more stable in solution according to findings on *Azotobacter vinelandii* AnfA and NifA, and *Herbaspirillum seropedicae* NifA (Austin & Lambert, 1994; Lee et al., 1993; Monteiro, Souza, Yates, Pedrosa, & Chubatsu, 2003).

AnfA_DBD bound the F1 fragment (carrying the wild‐type *anfH* promoter) demonstrating that the isolated C‐terminal domain indeed functions in promoter binding independent of the other AnfA domains (Figure 3c). Mut1 was bound comparably well as F1 indicating that this sequence is dispensable for AnfA binding. AnfA_DBD also bound promoter variants Mut2 to Mut7 albeit less effectively than F1, and the combined Mut2/6 and Mut2/7 variants were barely shifted. These observations suggest that recognition of the distal or proximal sites by AnfA is essentially unbiased. Taken together, the in vitro binding studies (Figure 3c) are well in line with the in vivo data on *anfH* expression (Figure 3b) supporting the hypothesis that the two 17 bp repeat sequences (Figure 2a) serve as distal and proximal AnfA‐binding sites.

### 3.4 NifA‐driven *anfHDGKOR* expression restores Fe‐nitrogenase activity in a strain lacking AnfA

Productive nitrogen fixation by the Fe‐nitrogenase requires more than the expression of the *anfHDGKOR3* operon. Current knowledge suggests that AnfA is required only for *anfHDGKOR3* expression and has little impact on the expression of NifA‐activated genes like *nifB* and *rnfA*, which are essential for FeFeco biosynthesis and electron transfer to Fe‐nitrogenase, respectively (Demtröder, Pfänder, et al., 2019; Schüddekopf et al., 1993). We, therefore, speculated that AnfA might be dispensable for the production of active (N_2_‐fixing) Fe‐nitrogenase as long as the *anfHDGKOR3* operon is adequately expressed. To achieve AnfA‐independent *anfHDGKOR3* expression, we replaced the *anfH* promoter (P*_anfH_*) by the NifA‐activated *nifH* promoter (P*_nifH_*). Both promoters mediate comparably strong expression of their downstream genes at least under Mo‐limiting conditions (Demtröder, Pfänder, et al., 2019). If the *anfHDGKOR3* operon is the only member of the AnfA regulon, nitrogen fixation by Fe‐nitrogenase should become entirely NifA‐dependent in the P*_anfH_* → P*_nifH_* strain (Figure 1b).

To determine the effect of P*_anfH_* → P*_nifH_* substitution on *anfHDGKOR3* expression, we generated two *R. capsulatus* strains, which carry the same P*_anfH_* → P*_nifH_* promoter substitution, but either in the wild‐type (*anfA* ^+^) or Δ*anfA* background. Subsequently, we introduced transcriptional *anfH*‐*lacZ* reporter fusions in these strains and, as a control, in the wild‐type strain B10S by chromosomal integration of plasmid pMH187 as previously described (Demtröder, Pfänder, et al., 2019). The resulting reporter strains were grown with either serine or ammonium as a nitrogen source before LacZ activity was determined.

**FIGURE 4:**
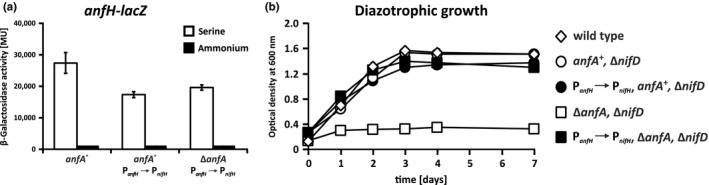
Effect of P*_anfH_* → P*_nifH_* substitution on *anfH*‐*lacZ* expression and diazotrophic growth. (a) Effect of P*_anfH_* → P*_nifH_* substitution on *anfH*‐*lacZ* expression. *R. capsulatus* strains carrying a chromosomally integrated transcriptional *anfH*‐*lacZ* fusion based on plasmid pMH187 (Demtröder, Pfänder, et al., 2019) were phototrophically grown in RCV minimal medium (no Mo added) with either 10 mM serine or 10 mM ammonium. The strains used were as follows: B10S:pMH187 (*anfA* ^+^, *anfH*‐*lacZ*), YP516:pMH187 (P*_anfH_* → P*_nifH_*, *anfA* ^+^, *anfH*‐*lacZ*), and YP515:pMH187 (P*_anfH_* → P*_nifH_,* Δ*anfA*, *anfH*‐*lacZ*). LacZ (β‐galactosidase) activity is given in Miller units (Miller, 1972). The results represent the means and standard deviations of five independent experiments. (b) Effect of P*_anfH_* → P*_nifH_* substitution on Fe‐nitrogenase activity in *R. capsulatus* strains lacking AnfA. *R. capsulatus* strains were phototrophically grown in RCV minimal medium (no Mo added) with N_2_ as the sole nitrogen source. The strains used were as follows: B10S (wild type), BS85 (*anfA* ^+^, Δ*nifD*), YP516‐BS85 (P*_anfH_* → P*_nifH_*, *anfA* ^+^, Δ*nifD*), YP515‐BS85 (P*_anfH_* → P*_nifH_*, Δ*anfA*, Δ*nifD*), and KS94A‐YP415 (Δ*anfA*‐Δ*nifD*). The results represent the means and standard deviations of three independent measurements

The wild‐type control (containing the native AnfA‐dependent *anfH* promoter) showed the expected high *anfH*‐*lacZ* expression in serine cultures, while no expression was observed in ammonium cultures (Figure 4a; Demtröder, Pfänder, et al., 2019; Kutsche et al., 1996; Wiethaus, Wirsing, Narberhaus, & Masepohl, 2006). Both P*_anfH_* → P*_nifH_* strains expressed *anfH*‐*lacZ* to comparable levels as the wild‐type control showing that P*_anfH_* → P*_nifH_* substitution mediated effective *anfHDGKOR3* expression independent of AnfA.

To test whether nitrogen fixation by Fe‐nitrogenase was entirely NifA‐dependent in the P*_anfH_* → P*_nifH_* strains, we introduced a polar *nifD* mutation (Δ*nifD*) in these strains, before examination of diazotrophic growth. Regardless of the *nifD* mutation, these strains were expected to express all the other NifA‐dependent nitrogen fixation genes involved in cofactor biosynthesis and electron transport (Figure 1b). Since Δ*nifD* strains lack Mo‐nitrogenase, diazotrophic growth of these strains depends entirely on Fe‐nitrogenase (Demtröder, Pfänder, et al., 2019). As controls, we included the wild type, the parental Δ*nifD* strain, and a Δ*anfA*‐Δ*nifD* strain lacking both nitrogenases.

The wild‐type and Δ*nifD* strains grew well with N_2_ as the sole nitrogen source, while the Δ*anfA*‐Δ*nifD* strain failed to grow diazotrophically (Figure 4b) consistent with earlier studies (Demtröder, Pfänder, et al., 2019; Kutsche et al., 1996). Both P*_anfH_* → P*_nifH_* strains grew almost as well as the parental Δ*nifD* strain suggesting that P*_anfH_* → P*_nifH_* substitution indeed decouples production of functional Fe‐nitrogenase from AnfA.

Taken together, our findings show that substitution of the AnfA‐dependent *anfH* promoter by the NifA‐activated *nifH* promoter restores *anfHDGKOR* expression and Fe‐nitrogenase activity in a strain lacking AnfA. In other words, AnfA appears to be dispensable for FeFeco biosynthesis and electron delivery to Fe‐nitrogenase.

## 4 DISCUSSION

Despite the wide distribution of Fe‐nitrogenases (McRose et al., 2017), our knowledge of Fe‐nitrogenase‐related promoters was so far limited to one species, the γ‐proteobacterium *Azotobacter vinelandii* (Austin & Lambert, 1994; Drummond et al., 1996). Here, we characterized two AnfA‐binding sites in the *anfH* promoter in the α‐proteobacterium *Rhodobacter capsulatus* by in vivo and in vitro studies. Each of these binding sites mediated *anfH* expression to some extent, but maximal expression required both AnfA‐binding sites.

**FIGURE 5:**
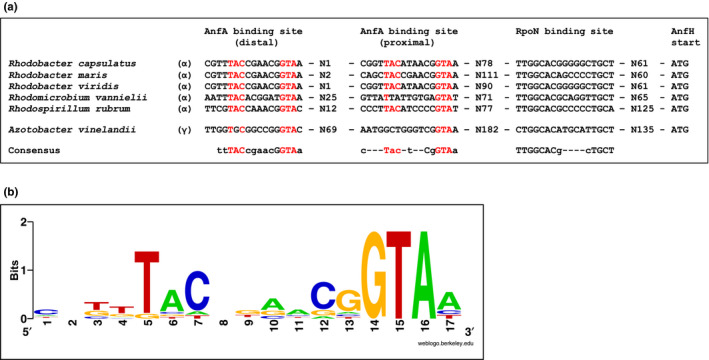
AnfA‐binding sites in proteobacterial *anfH* promoters. (a) Comparison of AnfA‐binding sites. Binding of AnfA to distal and proximal sites has been experimentally shown for *R. capsulatus* (this study) and *A. vinelandii* (Austin & Lambert, 1994). Affiliation of bacterial strains to the α‐ and γ‐proteobacteria, and the numbers of nucleotides (N) between cis‐regulatory elements are indicated. Known and presumed AnfA‐binding sites encompass strictly conserved GTA and partially conserved TAC motifs (highlighted in red). Lower and upper case lettering in the consensus sequences indicates conservation in at least four or five of the respective sequences, respectively. (b) AnfA‐binding site logo. The AnfA‐binding site consensus based on all distal and proximal sites shown in (a) was generated using the weblogo.berkeley.edu program

Sequences similar to the *R. capsulatus* AnfA‐binding sites are conserved in α‐proteobacterial promoters preceding potential *anfHDGK* operons (Figure 5a; McRose et al., 2017). Sequence logo representation (based on all distal and proximal AnfA‐binding sites shown in Figure 5a) revealed a conserved sequence of dyad symmetry, TAC–N_6_–GTA, as the AnfA‐binding site consensus (Figure 5b). Binding sites of dyad symmetry are typically bound by dimeric regulators (Kim & Little, 1992; Klose, North, Stedman, & Kustu, 1994) suggesting that AnfA proteins also bind target promoters as dimers. The AnfA‐binding site consensus previously defined for *A. vinelandii*, C–N–GG–N_3_–GGTA (Austin & Lambert, 1994), and our consensus share the strictly conserved GTA motif (Figure 5a). The *A. vinelandii* AnfA‐binding sites, which were identified by footprint experiments, lack the TAC motif indicating that this motif is dispensable for promoter recognition by *A. vinelandii* AnfA. In *R. capsulatus*, individual TAC (Mut2, Mut5) and GTA mutations (Mut4, Mut7) reduced *anfH*‐*lacZ* expression by 60%–80%, while the Mut2/Mut7 combination abolished expression (Figure 3b). Together these findings suggest that AnfA proteins in different bacteria require the GTA motif, but differ in their dependence on the TAC motif to activate their target promoters.

*Rhodobacter capsulatus* AnfA is essential for *anfHDGKOR3* expression and consequently, for nitrogen fixation by Fe‐nitrogenase (Demtröder, Pfänder, et al., 2019). Remarkably, a strain lacking AnfA but driving *anfHDGKOR3* expression by NifA regained the capacity to grow diazotrophically via Fe‐nitrogenase (Figure 4b). This means that AnfA in the wild type is required for AnfHDGKOR3 production, but dispensable for FeFeco biosynthesis and electron delivery to Fe‐nitrogenase. For a regulatory model, see Figure 1b. Our findings do not necessarily exclude other AnfA targets than the *anfH* promoter. Indeed, AnfA affects the expression of different nitrogen fixation genes including *iscN*, *nifE*, *fprA*, and *nifB* (Demtröder, Pfänder, et al., 2019). None of these genes, however, is preceded by an obvious AnfA‐binding site (or just a GTA motif) suggesting that AnfA control of these genes is indirect.

In contrast to the *R. capsulatus nifB* promoter, we found potential AnfA‐binding sites in the *nifB* promoters of *Rhodospirillum rubrum* (TAC–N_6_–GTA) and *Rhodomicrobium vannielii* (CAC–N_6_–GTA) suggesting direct *nifB* activation by AnfA in these strains. In line with the requirement of NifB for the activity of all three nitrogenases in *A. vinelandii*, the *nifB* promoter can be activated by NifA, VnfA, or AnfA, which bind to overlapping sites in the *nifB* promoter (Drummond et al., 1996).

Our finding that only a single Fe‐nitrogenase‐related target, the *anfH* promoter, strictly requires activation by AnfA in *R. capsulatus*, raises the question of why this diazotroph needs AnfA. One explanation is that AnfA contributes (indirectly) to fine regulation of NifA‐dependent genes (Demtröder, Pfänder, et al., 2019). Also, Mo repression of *anfA* introduces a regulatory level to prevent the production of Fe‐nitrogenase under Mo‐replete conditions. This guarantees the exclusive activity of Mo‐nitrogenase, which exhibits higher N_2_‐reducing activity than Fe‐nitrogenase (Hoffmann, Wagner, et al., 2016; Wiethaus et al., 2006).

## CONFLICT OF INTERESTS

None declared.

## AUTHOR CONTRIBUTION

**Lisa Demtröder:** Conceptualization (supporting); Investigation (lead); Writing‐original draft (equal). **Yvonne Pfänder:** Investigation (supporting). **Bernd Masepohl:** Conceptualization (lead); Funding acquisition (lead); Writing‐original draft (equal). 

## ETHICS STATEMENT

None required.

## Data Availability

All data are provided in full in the Section 3.

## APPENDIX 1

**TABLE A1:** *Rhodobacter capsulatus* strains and plasmids

Strain or plasmid	Relevant characteristics	Source or reference
Strains		
B10S	*R. capsulatus* wild type; Sm^r^	Klipp, Masepohl, and Pühler (1988)
BS85	*nifD*::Sp mutant (Δ*nifD*) of B10S; Sm^r^, Sp^r^	Hoffmann et al. (2014)
KS94A	*anfA*::Sp mutant (Δ*anfA*) of B10S; Sm^r^, Sp^r^	Wang, Angermüller, and Klipp (1993)
KS94A‐YP415	*anfA*::Sp, *nifD*::Gm mutant (Δ*anfA*, Δ*nifD*) of B10S; Gm^r^, Sm^r^, Sp^r^	This study
YP515‐BS85	P*_anfH_* → P*_nifH_*, Δ*anfA*::Gm^r^, Δ*nifD*::Sp^r^ mutant of B10S; Gm^r^, Sm^r^, Sp^r^	This study
YP516‐BS85	P*_anfH_* → P*_nifH_*, *anfA* ^+^, Δ*nifD*::Sp^r^ mutant of B10S; Gm^r^, Sm^r^, Sp^r^	This study
Vector plasmids		
pASK_IBA45+	AHT‐inducible expression vector; Ap^r^	IBA GmbH Göttingen, Germany
pBBR1MCS	Mobilizable broad‐host‐range vector; Cm^r^	Kovach et al. (1995)
pUC18	Narrow‐host‐range vector; Ap^r^	Norrander, Kempe, and Messing (1983)
pYP35	*lac*TeT cassette donor; Ap^r^, Te^r^, oriT	Gisin et al. (2010)
Hybrid plasmids		
pBBR_F1‐*lacZ* to pBBR_F6‐*lacZ*	pBBR1MCS derivatives carrying P*_anfH_* fragments F1 to F6 (Figure 2a,b) fused to *lacZ*; Cm^r^, Te^r^	This study
pBBR_Mut1‐*lacZ* to pBBR_Mut7‐*lacZ*; pBBR_Mut2/6‐*lacZ*; pBBR_Mut2/7‐*lacZ*	pBBR_F1‐*lacZ* variants carrying mutations Mut1 to Mut7, Mut2/6, and Mut2/7 (Figure 3a); Cm^r^, Te^r^	This study
pMH187	Mobilizable narrow‐host‐range plasmid carrying *anfH*‐*lacZ*; Te^r^, oriT	Demtröder, Pfänder, et al. (2019)
pYP409	pASK_IBA45 + derivative encoding AnfA_DBD; Ap^r^	This study
pYP515	pUC18 derivative carrying *rcc00583*‐Gm^r^‐P*_nifH_*‐*anfH*; Ap^r^, Te^r^, oriT	This study
pYP516	pUC18 derivative carrying *anfA*‐Gm^r^‐P*_nifH_*‐*anfH*; Ap^r^, Te^r^, oriT	This study

Abbreviations: Ap, ampicillin; Cm, chloramphenicol; Gm, gentamicin; Sm, streptomycin; Sp, spectinomycin; Te, tetracycline.

## APPENDIX 2

The figure shows the alignment of AnfA proteins from *Rhodobacter capsulatus* (Rcap; WP_023913804), *Rhodobacter maris* (Rmar; WP_097070194), *Rhodobacter viridis* (Rvir; WP_110806348), *Rhodomicrobium vannielii* (Rvan; WP_013420907), *Rhodospirillum rubrum* (Rrub; WP_011389142), and *Azotobacter vinelandii* (Avin; WP_012703363). Amino acid residues identical in all sequences are marked by stars. Underlined regions in the *A. vinelandii* protein indicate conserved motifs and domains, namely a cysteine motif (residues 21–26), the GAF domain (residues 33–193), the AAA + domain (residues 218–459), and the DNA‐binding helix‐turn‐helix domain (residues 482–522; adapted from Austin & Lambert, 1994; Joerger et al., 1989). The separated DNA‐binding domain of *R. capsulatus* AnfA (AnfA_DBD) used in electrophoretic mobility shift assays (Figure 3c) is marked in yellow.
